# Development of Mushroom-Based Cosmeceutical Formulations with Anti-Inflammatory, Anti-Tyrosinase, Antioxidant, and Antibacterial Properties

**DOI:** 10.3390/molecules21101372

**Published:** 2016-10-14

**Authors:** Oludemi Taofiq, Sandrina A. Heleno, Ricardo C. Calhelha, Maria José Alves, Lillian Barros, Maria Filomena Barreiro, Ana M. González-Paramás, Isabel C. F. R. Ferreira

**Affiliations:** 1 Mountain Research Centre (CIMO), ESA, Polytechnic Institute of Bragança, Campus de Santa Apolónia, 1172, 5300-253 Bragança, Portugal; taofiq.oludemi@ipb.pt (O.T.); sheleno@ipb.pt (S.A.H.); callelha@ipb.pt (R.C.C.); lillian@ipb.pt (L.B.); 2 GIP-USAL, Unidad de Nutrición y Bromatología, Faculty of Pharmacy, University of Salamanca, Campus Miguel de Unamuno, 37007 Salamanca, Spain; paramas@usal.es; 3 Laboratory of Separation and Reaction Engineering (LSRE), Associate Laboratory LSRE/LCM, Polytechnic Institute of Bragança, Campus de Santa Apolónia, 1134, 5301-857 Bragança, Portugal; barreiro@ipb.pt; 4 School of Health, Polytechnic Institute of Bragança, Av. D. Afonso V, 5300-121 Bragança, Portugal; maria.alves@ipb.pt

**Keywords:** cosmeceutical formulation, skin aging, mushrooms extracts, anti-inflammatory, anti-tyrosinase, antioxidant, antibacterial

## Abstract

The cosmetic industry is in a constant search for natural compounds or extracts with relevant bioactive properties, which became valuable ingredients to design cosmeceutical formulations. Mushrooms have been markedly studied in terms of nutritional value and medicinal properties. However, there is still slow progress in the biotechnological application of mushroom extracts in cosmetic formulations, either as antioxidants, anti-aging, antimicrobial, and anti-inflammatory agents or as hyperpigmentation correctors. In the present work, the cosmeceutical potential of ethanolic extracts prepared from *Agaricus bisporus*, *Pleurotus ostreatus*, and *Lentinula edodes* was analyzed in terms of anti-inflammatory, anti-tyrosinase, antioxidant, and antibacterial activities. The extracts were characterized in terms of phenolic acids and ergosterol composition, and further incorporated in a base cosmetic cream to achieve the same bioactive purposes. From the results obtained, the final cosmeceutical formulations presented 85%–100% of the phenolic acids and ergosterol levels found in the mushroom extracts, suggesting that there was no significant loss of bioactive compounds. The final cosmeceutical formulation also displayed all the ascribed bioactivities and as such, mushrooms can further be exploited as natural cosmeceutical ingredients.

## 1. Introduction

Skin aging is caused by an intrinsic or natural mechanism affecting the skin and other body organs related to hormonal changes occurring with age, and by extrinsic mechanisms associated with ultraviolet radiation exposure, which causes free radical species generation [1]. Reactive oxygen species (ROS) are responsible for inducing the activator protein-1 (AP-1), a transcription factor that promotes collagen and elastin breakdown by up regulation of the matrix metalloproteinases (MMPs) [2]. This environmental stress generated from ROS and reactive nitrogen species (RNS), combined with increased secretion of inflammatory mediators and enzyme expression (e.g., collagenase and elastase), causes inflammation and decreases the tensile strength and elasticity of the skin [3]. Also in the antimicrobial domain, the incidence of skin and soft tissue infections is growing as a consequence of increasing bacteria resistance to drugs in a wide clinical spectrum [4].

Natural ingredients for skin care are becoming popular due to their protective and defensive role against generation of free radicals and reduced production of oxidative enzymes, such as inducible nitric oxide synthase (iNOS) associated with inflammation and inflammatory diseases; collagenase and elastase that usually cause degradation of the extracellular matrix of the skin; and tyrosinase, the enzyme that catalyzes the most important step in melanin biosynthesis [5]. Thus, an increasing interest in finding natural sources able to inhibit these enzymes, and using them as potential cosmetic ingredients in the design of creams or lotions for topical application is an interesting topic [6].

Cosmeceutical formulations containing bioactive ingredients—such as phytonutrients, microbial metabolites, dairy products, minerals, and animal proteins—displaying medical-drug like benefits able to improve the structure, function, and appearance of the skin, are becoming more and more appellative solutions [7].

Particularly, mushrooms, which are recognized as nutritionally important foods and reported as having important medicinal benefits, are slowly finding their way into the cosmetic industry in cosmeceutical applications, either as creams, lotions, or ointments [7]. *Agaricus bisporus* (J.E.Lange) Imbach, *Lentinula edodes* (Berk.) Pegler, and *Pleurotus* species are the most cultivated mushrooms worldwide, with a commercial production projected to increase significantly in the coming years [8]. *A. bisporus* has been known to show medicinal potential related with antitumor, antimicrobial, immunomodulatory, anti-inflammatory, and antioxidant properties [9]. *L. edodes,* popularly known as “shiitake”, has been reported to halve the proliferation of tumor cells and to strengthen the immune system [10]. This mushroom has several bioactive compounds such as l-ergothioneine and lentinan known to exert strong antioxidant [11,12] and anti-inflammatory [13] activities, respectively. *Pleurotus ostreatus* (Jacq. ex Fr.) P. Kumm., has also attracted attention due to its content in polysaccharides, proteins, organic acids, vitamins, and minerals [14], being reported as having anti-inflammatory [15], antioxidant [16], immunomodulatory, antimicrobial, and antitumor effects [17]. All these aforementioned medicinal properties of mushroom extracts and related bioactive constituents, reinforce the interest in these natural matrices for cosmetic applications.

Some mushroom-based cosmetics can be found in the market; examples include *Aveeno Positively Ageless*, which contains shitake (*L. edodes*) extract (www.aveeno.com) used to improve skin’s health; *Sekkisei Cream* containing *Cordyceps sinensis* (Berk.) Sacc., extract (www.coracosmetics.com), used to strengthen skin’s natural defense system and to suppress melanin production, thereby correcting hyperpigmentation; *Embellir Refresh Massage* that contains *Ganoderma lucidum* (Curtis) P. Karst extract (www.menard-cosmetic.com) used as a cleansing and massage cream and also as an efficient anti-aging skin care product.

The present study aimed to evaluate the cosmeceutical potential of *A. bisporus*, *L. edodes*, and *P. ostreatus* ethanolic extracts through the assessment of their anti-inflammatory, anti-bacterial, antioxidant, and anti-tyrosinase activities, and further incorporation in a cosmetic base cream. The bioactive properties of the final formulations were confirmed and related with the chemical ingredients present in the extracts, namely with phenolic acids and ergosterol.

## 2. Results and Discussion

### 2.1. Chemical Characterization of the Mushroom Ethanolic Extracts

The chemical composition of the mushroom extracts is presented in Table 1. *P. ostreatus* showed the highest content in phenolic acids and cinnamic acid (related compound), followed by *L. edodes* and, finally, *A. bisporus.* The highest content in *P. ostreatus* is due to the contribution of cinnamic and *p*-hydroxybenzoic acids, found in much higher amounts in this species, followed by *p*-coumaric acid. Protocatechuic acid was only identified in *L. edodes*, while *A. bisporus* only revealed the presence of cinnamic acid.

The results are consistent with the ones published by Taofiq et al. [15] that reported the presence of all those three phenolic acids in *P. ostreatus* ethanolic extract obtained by maceration and only cinnamic acid in *A. bisporus*. Carneiro et al. [18] described the presence of vanillic and *p*-coumaric acids in methanol/water (80:20, *v**/**v*) extract of *L. edodes* from Brazil. The differences between the used extraction procedures and mushroom origin can justify the dissimilarity observed in the achieved phenolic acids profile.

In the present study, the magnitude of the ergosterol content was: *P. ostreatus* > *A. bisporus* > *L. edodes*, even though Barreira et al. [19] reported a different order—namely, *A. bisporus* > *L. edodes* > *P. ostreatus*—but these authors used Soxhlet extraction with a different solvent (*n*-hexane).

### 2.2. Bioactive Properties of the Mushroom Ethanolic Extracts

#### 2.2.1. Anti-Inflammatory Activity

The anti-inflammatory effects of the ethanolic extracts were assessed upon stimulation of RAW 264.7 macrophages with lipopolysaccharide for the production of the inflammatory mediator (NO). The most efficient species were *A. bisporus* and *L. edodes,* presenting the lowest EC_50_ values, followed by *P. ostreatus* (Table 2). The high activity exhibited by *L. edodes* extract may be related with its high content of protocatechuic acid, but without discarding the presence of other metabolites from different chemical classes such as terpenes, polysaccharides, and lipid metabolites that have also been reported as important contributors to the anti-inflammatory activity of mushroom extracts. The base cream was evaluated for anti-inflammatory activity and up to 800 μg/mL, the base cream did not display activity.

The anti-inflammatory potential of different mushroom extracts has been described and related to its capacity to inhibit specific steps in the pathway leading to nuclear factor kappa B (NF-κB) release [20]. Taofiq et al. [15] reported that *P. ostreatus* ethanolic extract had a better anti-inflammatory activity than *A. bisporus* but using a different extraction methodology (maceration).

#### 2.2.2. Anti-Tyrosinase Activity

*A. bisporus* displayed the highest anti-tyrosinase activity among the studied mushroom species (Table 2). *P. ostreatus* and *L. edodes* exhibited very similar activities. Alam et al. [21] reported that the methanolic extract of *P. ostreatus*, at 0.125–1 mg/mL, significantly inhibited tyrosinase activity up to 11.36%–59.56%. To the author’s best knowledge, there are no reports on the anti-tyrosinase activity of *A. bisporus*; nevertheless, Miyake et al. [22] described that 2-amino-3*H*-phenoxazin-3-one, a phenolic compound isolated from the fruiting body of this mushroom species, at 0.5 μM, significantly inhibited tyrosinase activity up to 80%. Also, Yoon et al. [23] described that the methanolic extract of *L. edodes* at 0.125–1 mg/mL, significantly inhibited tyrosinase activity by 15.12%–54.61%. The base cream was evaluated for anti-tyrosinase activity and, up to 2 mg/mL, the base cream did not inhibit tyrosinase enzyme.

Phenolic compounds have been reported to be responsible for the anti-tyrosinase activity of mushroom extracts, but Yan et al. [24] also isolated two steroidal triterpenes (betulin and trametenolic acid) from *Inonotus obliquus* (Ach. ex Pers.) Pilát and described their anti-tyrosinase activity. Betulin significantly inhibited tyrosinase activity with an IC_50_ of 5.13 μM, being even stronger than kojic acid (6.43 μM) used as control, while trametenolic acid had an IC_50_ of 7.25 μM. The activity displayed by the studied mushroom extracts were related with the identified phenolic acids and ergosterol.

Tyrosinase is the rate-limiting enzyme in the melanin biosynthesis pathway where it converts tyrosine to dihydroxyphenylalanine (DOPA) and further oxidizes DOPA to dopaquinone [25]. Several factors such as exposure to UV radiation and release of α-melanocyte-stimulating hormone trigger over-secretion of melanin from melanocytes causes hyperpigmentation [26]. Finding inhibitors of tyrosinase from natural sources is a topic of research interest and the studied mushroom extracts can be considered good candidates.

#### 2.2.3. Antioxidant Activity

*A. bisporus* and *P. ostreatus* showed the highest radical scavenging activity and reducing power (Table 2). The obtained results are consistent with the ones reported by Reis et al. [27], where *A. bisporus* methanolic extracts obtained by maceration gave the highest DPPH radical scavenging activity. Heleno et al. [28] reported that the reducing power of *L. edodes* methanolic extracts obtained by maceration revealed an EC_50_ of 3.19 ± 0.03 mg/mL, similar to the one reported in the present study. The base cream was also evaluated for antioxidant activity and, up 200 mg/mL, the base cream did not display DPPH radical scavenging activity or reducing power.

Finding extracts or bioactive metabolites that display antioxidant activity seems to be one of the most important parameters in the design of cosmeceutical formulations, not just because they scavenge ROS but also because they are able to inhibit tyrosinase and matrix metalloproteinase enzymes responsible for hyperpigmentation and collagen degradation, respectively [29].

#### 2.2.4. Antibacterial Activity

The resistance profiles to antibiotics of all tested bacteria strains are presented in Supplementary Materials for Gram negative and Gram positive bacteria. All the mushroom extracts showed antibacterial potential against MSSA and MRSA (Table 3). Regarding the Gram-positive bacteria strains, in general, *L. edodes* showed the highest activity against the tested bacteria presenting the lowest MIC values, while *A. bisporus* revealed the weakest activity presenting the highest MIC values against MSSA and MRSA and no activity against *E. faecalis*, *P. ostreatus*, and *L. edodes* showed antibacterial activity against *E. faecalis*. None of the extracts was able to inhibit the growth of Gram-negative bacteria strains, even at the highest tested concentration (20 mg/mL). These results are in agreement with the ones reported by Barros et al. [30] that described the absence of antibacterial activity of *A. bisporus* methanolic extracts against *P. aeruginosa*. Alves et al. [31] also reported no activity for *A. bisporus* against resistant *P. aeruginosa* and *E. coli*. However, Stojković et al. [9] reported antibacterial activity of this mushroom (methanolic and ethanolic extracts) against *E. coli* and *P. aeruginosa*, but this may be due to the fact that these authors used ATCC bacteria whereas, in the present work, all the bacteria are clinical isolates with antibiotics resistance profile. The base cream was evaluated for antibacterial activity and, up 200 mg/mL, no activity was observed against all the tested bacteria.

Several authors have reported that the normal microflora of the skin include *Staphylococcus epidermis*, *S. aureus*, and *P. aeruginosa*, among others [32]. Taking into account that these microorganisms are opportunistic and have high resistance profiles, they are a threat when skin lesions occur, causing severe localized infections even with systemic invasion [33].

Thus, finding extracts/compounds with high antibacterial activity against these species can be considered beneficial in the design of cosmeceutical formulations. The mechanism of phenolic compounds’ antibacterial activity has been attributed to its ability to interfere with the bacteria membrane causing their disruption and subsequent DNA damage [34].

### 2.3. Chemical Profile and Bioactivity of the Final Cosmeceutical Formulations

In the base creams incorporated with the extracts, the presence of phenolic acids, cinnamic acid, and ergosterol was monitored by HPLC-PDA. By analyzing Table 1, it can be concluded that all the target compounds were stable when incorporated in the base cream matrix. In fact, they were detected at almost the same amount as initially found in the extracts. It was possible to quantify in the final cosmeceutical formulations 85%–100% of the content quantified in the extracts before incorporation. The total phenolic acids found in *A. bisporus*, *P. ostreatus*, and *L. edodes* extract was 90.06 ± 0.74, 584.24 ± 3.01, and 142.81 ± 2.39 μg/g, respectively, while after incorporation in the base cosmetic cream, the total phenolic acids became 87.73 ± 1.63, 509.47 ± 3.93, and 119.61 ± 2.54 (μg/g), respectively, suggesting that there was no significant loss of bioactive compounds. The same trend was observed for the ergosterol content in the mushroom extracts after incorporation.

The anti-inflammatory, anti-tyrosinase, antioxidant, and antibacterial activities of the final cosmeceutical formulations were also evaluated and the results are given in Table 2. The final formulations incorporated with *A. bisporus* and *L. edodes* showed the highest anti-inflammatory activity like it was observed with the individual ethanolic extracts; while *P. ostreatus* presented the lowest anti-inflammatory potential. The cosmeceutical formulation incorporated with *A. bisporus* extract revealed the highest anti-tyrosinase, while *P. ostreatus* and *L. edodes* revealed the lowest one, also in agreement with the individual activity of the extracts before incorporation. *A. bisporus* and *P. ostreatus* cream formulations showed the highest antioxidant activity, while *L. edodes* presented the lowest DPPH scavenging activity and reducing power.

All the cosmeceutical formulations showed inhibition against MSSA and MRSA (Table 3). *A. bisporus* cream formulation revealed the lowest activity for MSSA and MRSA, and did not present activity against *E. faecalis*. *L. edodes* cream formulation showed the highest activity against *E. faecalis*, MSSA, and MRSA. The cosmeceutical formulations displayed antibacterial activity against the same bacteria species that were inhibited by the extracts.

All the bioactivities were affected when extracts were incorporated into the cream formulations, increasing the EC_50_ values to higher concentrations, but still displaying all the bioactivities evaluated and maintaining the levels of the analyzed bioactive compounds. This increase in the EC_50_ values can be due to some interferences of the base cream that may affect the extract availability to exert the same bioactivity. Considering the antioxidant and the antibacterial activities exhibited by the final cream formulations, mushroom extracts can also act as preservatives in the final formulations.

## 3. Materials and Methods

### 3.1. Standards and Reagents

Acetonitrile 99.9% was of high-performance liquid chromatography (HPLC) grade from Lab-Scan (Lisbon, Portugal), methanol was of analytical grade and supplied by Pronalab (Lisbon, Portugal). 2,2-Diphenyl-1-picrylhydrazyl (DPPH) was obtained from Alfa Aesar (Ward Hill, MA, USA). Dulbecco’s modified Eagle’s minimum essential medium (DMEM), fetal bovine serum (FBS), penicillin, streptomycin, Griess reagent system (Promega), DMSO, sulphorodamine B (SRB), lipopolysaccharide (LPS), 3,4-dihydroxy-l-phenylalanine (l-DOPA), and mushroom tyrosinase enzyme were obtained from Sigma-Aldrich Co. (Saint Louis, MO, USA). The culture media Muller Hinton broth (MHB) and Tryptic Soy Broth (TSB) were obtained from Biomerieux (Marcy l’Etoile, France). Blood agar with 7% sheep blood and Mac Conkey agar plates were purchased from Biomerieux Marcy l’Etoile, France). The dye *p*-iodonitrotetrazolium chloride (INT) was purchased from Sigma-Aldrich (Spruce Street; St. Louis, MO, USA) and was used as microbial growth indicator.

### 3.2. Mushroom Species and Preparation of the Ethanolic Extracts

Fresh samples of *Agaricus bisporus* (J.E.Lange) Imbach, *Lentinula edodes* (Berk.) Pegler, and *Pleurotus ostreatus* (Jacq. ex Fr.) P. Kumm) were purchased from a local supermarket in the Northeast of Portugal. For each mushroom species, the fruiting bodies were dried at 30 °C in an oven, reduced to a fine powder (20 mesh) and the treated portions mixed together to obtain homogeneous samples for further use.

To obtain the extracts, 3.0 g of the dried powder was mixed with 150 mL of ethanol and extracted in a Soxhlet apparatus for 4 h (12 cycles). Finally, the solvent was evaporated under reduced pressure (rotary evaporator Büchi R-210, Flawil, Switzerland) to obtain the dried ethanolic extracts [35].

### 3.3. Chemical Characterization of the Extracts

#### 3.3.1. Analysis of Phenolic Acids

Dried extracts were re-dissolved in a water/ethanol mixture (80:20, *v*/*v*) at a concentration of 20 mg/mL and filtered through a 0.22 μm nylon disposable filter. The Shimadzu 20A series ultra-fast liquid chromatograph (UFLC, Shimadzu Corporation, Kyoto, Japan), operating at 0.5 mL/min and equipped with a Waters Spherisorb S3 ODS2 C18 column (3 μm, 150 × 4.6 mm) thermostatted at 35 °C, was used for phenolic acids identification and quantification. A binary solvent mixture consisting of 0.1% formic acid in water (A) and acetonitrile (B) was used. The detection was performed using a photodiode array detector (PDA) at 280 nm as the preferred wavelength [27]. The phenolic acids were quantified using calibration curves obtained from commercial standards, namely: protocatechuic acid (*y* = 164741*x*; *R*^2^ = 0.999), *p*-hydroxybenzoic acid (*y* = 113523*x*; *R*^2^ = 0.999), *p*-coumaric acid (*y* = 433521*x*; *R*^2^ = 0.998), and cinnamic acid (*y* = 583527*x*; *R*^2^ = 0.998). The results were expressed as μg per g of extract.

#### 3.3.2. Analysis of Ergosterol

Each dried mushroom extract was re-dissolved in methanol at a final concentration of 10 mg/mL and filtered through a 0.22 μm nylon disposable filter. The ergosterol identification and quantification was performed in HPLC-UV equipment, an integrated system composed by a pump (Knauer, Smartline system 1000, Berlin, Germany), a degasser system (Smartline manager 5000), an auto-sampler (AS-2057 Jasco, Easton, MD, USA), and a UV detector (Knauer Smartline 2500) according to a methodology developed in-house [19]. Chromatographic separation was achieved with an Inertsil 100A ODS-3 reversed-phase column (5 μm, 4.6 × 150 mm, BGB Analytik AG, Boeckten, Switzerland) operating at 35 °C (7971R Grace oven). The mobile phase was acetonitrile/methanol (70:30, *v*/*v*) and detection was performed at 280 nm. Ergosterol was quantified using a calibration curve obtained from a commercial standard (*y* = 0.71727*x* + 0.20476; *R*^2^ = 0.99624). The results were expressed as mg per g of extract.

### 3.4. Evaluation of the Anti-Inflammatory Activity

Anti-inflammatory activity was evaluated following the procedure reported by Taofiq et al. [15]. Briefly, the RAW 264.7 macrophage cell line is cultured in DMEM medium and supplemented with 10% heat-inactivated fetal bovine serum, glutamine, and antibiotics at 37 °C under 5% CO_2_, in humidified air. For each experiment, cells were detached with a cell scraper to achieve a cell density of 5 × 10^5^ cells/mL. Trypan blue dye exclusion test was used to estimate the proportion of dead cells that were found to be less than 5%. Cells were seeded in 96-well plates at 150,000 cells/well and allowed to stay overnight for proper attachment to the plate. Then, cells were treated with extract solutions (concentration of 400–50 μg/mL, for each extract) for 1h. Dexamethasone (50 μM) was used as a positive control. Then, macrophage cells with the extract were stimulated with LPS (1 μg/mL) for a period of 18 h. The effect of all the tested samples in the absence of LPS was also evaluated, in order to observe if they induced changes in nitric oxide (NO) basal levels. In negative controls, no LPS was added. The extracts and LPS were dissolved in supplemented DMEM.

Each ethanolic extract was dissolved in 50% DMSO at 8 mg/mL and submitted to successive dilutions (400 to 50 μg/mL). Griess reagent System kit which contains sulphanilamide, *N*-(1-napthyl) ethylenediamine hydrochloride (NED), and nitrite solutions was used to quantify nitric oxide. A reference curve of nitrite (sodium nitrite 100 μM to 1.6 μM; *y* = 0.0065*x* + 0.1309; *R*^2^ = 0.9988) was prepared in a 96-well plate. The cell culture supernatant (100 μL) was transferred to the plate and mixed with sulphanilamide and NED solutions, 5–10 min each, at room temperature. The produced nitric oxide was determined by measuring the absorbance at 540 nm (microplate reader ELX800 Biotek, Bio-Tek Instruments, Inc.; Winooski, VT, USA), and compared with the standard calibration curve. EC_50_ values were calculated from the graph reporting the percentage inhibition of NO production versus extract concentration.

### 3.5. Evaluation of the Anti-Tyrosinase Activity

Each ethanolic extract was dissolved in 50% DMSO at 10 mg/mL and submitted to successive dilutions (10 to 0.625 mg/mL). Tyrosinase inhibition assay was performed using L-DOPA as substrate according to the procedure described by Yoon et al. [36] with slight modifications. The assays were carried out in a 96-well microplate with each well containing 40 μL of the sample, 80 μL of phosphate buffer (0.1 M, pH 6.8), 40 μL of tyrosinase enzyme (60 units/mL), and 40 μL of l-DOPA (3.5 mM). The mixture was incubated for 10 min at 37 °C, and the absorbance measured at 475 nm using a microplate reader (ELX800 Bio-Tek Instruments, Inc.). l-ascorbic acid was used as the positive control and the results were compared with a control comprising 50% DMSO in place of the sample. The percentage of tyrosinase inhibition was calculated as follows: [(A_control_ − A_sample_)/A_control_] × 100. EC_50_ values were calculated from the calibration curve tyrosinase inhibition percentage versus extract concentration.

### 3.6. Evaluation of the Antioxidant Activity

#### 3.6.1. Reducing Power

Each ethanolic extract was dissolved in ethanol at 50 mg/mL and submitted to successive dilution (50 mg/mL to 1.56 mg/mL). The extract solutions with the different concentrations (0.5 mL) were mixed with sodium phosphate buffer (200 mmol/L, pH 6.6, 0.5 mL) and potassium ferricyanide (1% *w*/*v*, 0.5 mL). The mixture was incubated at 50 °C for 20 min, followed by addition of trichloroacetic acid (10% *w*/*v*, 0.5 mL). Then, 0.8 mL of the mixture was transferred to a 48-well plate, followed by addition of deionized water (0.8 mL), ferric chloride (0.1% *w*/*v*, 0.16 mL) and the absorbance measured at 690 nm in the Microplate Reader mentioned above [37]. The sample concentration providing 0.5 of absorbance (EC_50_ values) was calculated from the calibration curve absorbance (690 nm) versus extract concentrations. Trolox was used as positive control.

#### 3.6.2. DPPH Scavenging Activity

The prepared ethanolic extracts were dissolved in ethanol at 50 mg/mL and successive dilutions (50 mg/mL to 1.56 mg/mL) were made. For the reaction mixture, 30 μL of each extract solution and 270 μL of methanol containing DPPH radicals (6 × 10^−5^ mol/L) were added. The mixture was allowed to stand in the darkness for 1 h. The absorbance was further measured at 515 nm in the Microplate Reader mentioned above [37]. The radical scavenging activity (RSA) was calculated using the equation: %RSA = [(A_DPPH_ − A_S_)/A_DPPH_] × 100, where A_S_ is the absorbance of the sample solution with the sample and A_DPPH_ is the absorbance of the DPPH solution. The concentrations responsible for 50% of RSA (EC_50_ values) were calculated from the graphs of RSA percentages versus extract concentrations. Trolox was used as positive control.

### 3.7. Evaluation of the Antibacterial Activity

#### 3.7.1. Bacteria Strains

As multiresistant bacteria are more problematic due to their resistance to a wide range of antibiotics, clinical isolates were used instead of ATCC bacteria. Therefore, bacteria strains correspond to clinical isolates obtained from patients hospitalized in various departments at the Hospital Center of Trás-os-Montes and Alto Douro (Vila Real, Portugal). Two Gram-negative bacteria (*Escherichia coli* (isolated from urine), and *Pseudomonas aeruginosa* (isolated from expectoration)) and three Gram-positive bacteria (*Enterococcus faecalis* (isolated from urine), methicillin-sensitive *Staphylococcus aureus* (MSSA) (isolated from wound exudate), and methicillin-resistant *Staphylococcus aureus* (MRSA) (isolated from expectoration)) were tested. All the tested bacteria were placed to grow in the appropriate fresh medium, 24 h, and kept in the oven at 37 °C before analysis in order to maintain the exponential growth phase.

#### 3.7.2. Characterization of the Antibiotic Susceptibility of the Bacteria Strains

Microorganism identification and susceptibility tests were performed using MicroScan panels (MicroScan^®^; Siemens Medical Solutions Diagnostics, West Sacramento, CA, USA) by microdilution plate method. The interpretation criteria were based on Interpretive Breakpoints as indicated in Clinical and Laboratory Standards Institute (CLSI) [38]; Committee of L’Antibiogramme de la Société Française de Microbiologie (CA-SFM) [39]; and in the European Committee on Antimicrobial Susceptibility Testing (EUCAST) [40].

#### 3.7.3. Determination of the Minimal Inhibitory Concentration (MIC)

Each ethanolic extract was dissolved in 50% DMSO at 220 mg/mL. The determination of the minimal inhibitory concentration (MIC) was performed by the microdilution method and the rapid *p*-iodonitrotetrazolium chloride (INT) colorimetric assay according to [41] with some modifications. Initially, 50 μL of the extract solution in 50% DMSO at 220 mg/mL was added to 450 μL of medium TSB or MHB, according to the bacteria requirements, making a solution of 22 mg/mL at 5% DMSO. Then, 190 μL of this extract solution was added to each well of the 96-well microplate and therefore submitted to successive dilutions over the wells containing 90 μL of MHB or TSB media, starting by taking 100 μL of the higher concentration to the right below one and thus repeating this procedure sequentially. Afterwards, 10 μL of inoculum (1.5 × 10^8^ CFU/mL) was added to all the microplate wells thus achieving a concentration range of 20 to 0.156 mg/mL. Three negative controls were prepared (one with MHB/TSB 5% DMSO, another one with the extract, and the third one with medium and antibiotic). For the Gram negative bacteria, colistin was used at a concentration based on the obtained MIC (Table S1, Supplementary Materials); for the Gram positive bacteria, vancomycin was used (Table S2, Supplementary Materials). A positive control was also performed with MHB/TSB medium at 5% DMSO and the inoculum. The plates were then incubated at 37 °C, for 24 h, in an oven (Jouan, Berlin, Germany). The MIC of the samples was determined after adding INT (0.2 mg/mL, 40 μL) and after incubation at 37 °C for 30 min. Viable microorganisms reduced the yellow dye to a pink color. MIC was defined as the lowest extract concentration that prevented this change, thus showing complete inhibition of bacterial growth.

### 3.8. Development of the Cosmeceutical Formulations Based on Mushroom Extracts

The cosmetic cream used in this study is a commercial base cream purchased from Fagron Iberica S.A.U. (Barcelona, Spain). It is described as a bright, white to yellowish color; free from fragrances, colorants, parabens, mineral oils, sodium lauryl sulphate (SLS), propylene glycol, and ethoxylates; and composed of purified water, O/W emulsifiers, emollient, lubricant, pro-liposome, vitamin E, chelating agents, silicone, and preservatives. According to the supplied technical data, all ingredients are recognized as safe by the US Food and Drug Administration (FDA), Regulation (EC) No 1907/2006—REACH and the National Health Surveillance Agency (ANVISA) of Brazil. Considering the MIC values for the antibacterial activity, the highest ones were observed for *A. bisporus* and *P. ostreatus*, (~10 mg/mL). For *L. edodes*, the highest EC_50_ value was found for the antioxidant activity (~25 mg/mL). In order to cover all the bioactivities and considering the dilutions necessary to carry out the assays, the base creams were incorporated with 10 × MIC value for *A. bisporus* and *P. ostreatus* and 10 × EC_25_ value for *L. edodes*, which correspond to a scale of 100 mg of extract per gram of base cream. The base-cream and the extract were carefully mixed to guarantee sample homogeneity and analyzed immediately after incorporation to study the ability of the extracts to maintain their bioactivities in the cosmetic base cream.

The final cream formulations were extracted with methanol for 30 min, filtered, dried in a rotary evaporator, and re-dissolved in a water:methanol mixture (80:20, *v*/*v*) at 20 mg/mL for phenolic acids analysis; in methanol at 10 mg/mL for ergosterol analysis; in 50% DMSO at 200 mg/mL for anti-inflammatory activity evaluation; in 50% DMSO at 200 mg/mL for anti-tyrosinase activity evaluation; in 100% DMSO at 200 mg/mL for antioxidant activity evaluation; and in 5% DMSO at 220 mg/mL for antibacterial activity evaluation.

### 3.9. Statistical Analysis

For all the experiments, three independent samples were prepared and all the assays were carried out in triplicate. The results are expressed as mean values ± standard deviation (SD). The differences between the samples were analyzed using one-way analysis of variance (ANOVA) followed by Tukey’s honestly significant difference post hoc test with α = 0.05, coupled with Welch’s statistic. This treatment was carried out using IBM SPSS Statistics for Windows version 23.0 program (IBM Corp., Armonk, NY, USA).

## 4. Conclusions

Based on the above findings, it can be concluded that ethanolic extracts of *A. bisporus*, *P. ostreatus*, and *L. edodes* own strong antioxidant activity which suggest that they have the ability to cope with oxidative stress promoting ROS generation and acting as skin anti-aging. The extracts revealed antibacterial activity against MSSA and MRSA, which are microorganisms found to colonize the skin during injury and inflammation. After incorporating the extracts into the base cosmetic cream, the mushroom cosmetic formulations still display antioxidant and anti-inflammatory activity by inhibition of NO production and melanin by suppression of tyrosinase activity. Furthermore, the final cosmeceutical formulations containing the mushroom extracts were found to inhibit important bacteria strains responsible for skin damage. These diverse functions displayed by the mushroom extracts—before and after incorporation in the base cream formulations—suggest that mushrooms contain sustainable bioactive compounds that can be the basis of cosmeceutical formulations capable to tackle skin aging, inflammation, and hyperpigmentation. Hence, studies in dermal and epidermal cells should be conducted in order to clarify the role of the compounds responsible for the assessed bioactive properties. Further studies are currently being conducted regarding the evaluation of the stability of the cosmeceutical formulations.

## Figures and Tables

**Table 1:** Phenolic acids and ergosterol content in the mushroom ethanolic extracts and in the cosmeceutical formulations.

Phenolic Acids (μg/g)	Mushroom Extracts			Cosmeceutical Formulations		
	*A. bisporus*	*P. ostreatus*	*L. edodes*	*A. bisporus* cream	*P. ostreatus* Cream	*L. edodes* Cream
Cinnamic acid	90.06 ± 0.74 ^b^	362.7 ± 1.28 ^a^	7.31 ± 0.14 ^c^	87.73 ± 1.63 ^b^	317.43 ± 1.32 ^a^	6.10 ± 0.56 ^c^
*p*-Hydroxybenzoic acid	nd	157.78 ± 4.13	83.05 ± 2.15	nd	138.88 ± 2.30	73.67 ± 1.56
*p*-Coumaric acid	nd	63.74 ± 0.15	nd	nd	53.16 ± 2.94	nd
Protocatechuic acid	nd	nd	52.45 ± 0.38	nd	nd	39.85 ± 1.53
Total (μg/g)	90.06 ± 0.74 ^c^	584.24 ± 3.01 ^a^	142.81 ± 2.39 ^b^	87.73 ± 1.63 ^c^	509.47 ± 3.93 ^a^	119.61 ± 2.54 ^b^
Ergosterol (mg/g)	44.79 ± 0.37 ^b^	78.20 ± 0.54 ^a^	8.94 ± 0.04 ^c^	45.43 ± 1.38 ^b^	71.62 ± 0.29 ^a^	8.60 ± 0.25 ^c^

nd—not detected. In each row and within each group of samples (mushrooms extracts and final cosmeceutical formulations), different letters mean significant statistical differences (*p* < 0.05).

**Table 2:** Anti-inflammatory, anti-tyrosinase, and antioxidant activities of the mushroom ethanolic extracts and of the cosmeceutical formulations.

Bioactivities	Mushroom Extracts			Cosmeceutical Formulations		
	*A. bisporus*	*P. ostreatus*	*L. edodes*	*A. bisporus* Cream	*P. ostreatus* Cream	*L. edodes* Cream
Anti-inflammatory (EC_50_ value, mg/mL)	0.18 ± 0.01 ^b^	0.29 ± 0.03 ^a^	0.16 ± 0.01 ^b^	2.52 ± 0.23 ^b^	3.81 ± 0.23 ^a^	2.59 ± 0.23 ^b^
Anti-tyrosinase (EC_50_ value, mg/mL)	0.16 ± 0.01 ^b^	0.86 ± 0.07 ^a^	0.82 ± 0.08 ^a^	3.22 ± 0.37 ^b^	11.01 ± 0.35 ^a^	11.89 ± 0.85 ^a^
Antioxidant (EC_50_ value, mg/mL)						
DPPH radical-scavenging activity	7.04 ± 0.32 ^b^	7.69 ± 0.20 ^b^	23.36 ± 1.11 ^a^	234.3 ± 10.9 ^b^	239.5 ± 10.4 ^b^	321.8 ± 16.4 ^a^
Reducing power	2.34 ± 0.05 ^b^	2.36 ± 0.08 ^b^	3.03 ± 0.04 ^a^	35.91 ± 0.31 ^b^	32.18 ± 2.73 ^c^	48.90 ± 0.64 ^a^

Dexamethasone was used as positive control for anti-inflammatory activity (EC_50_ = 0.016 mg/mL). Ascorbic acid was used as positive control for anti-tyrosinase activity (EC_50_ = 0.031 mg/mL). Trolox was used as positive control for antioxidant activity (EC_50_ = 0.041 mg/mL for reducing power and 0.042 mg/mL for DPPH scavenging activity. In each row and within each group of samples (mushrooms extracts and final cosmeceutical formulations), different letters mean significant statistical differences (*p* < 0.05).

**Table 3:** Antibacterial activity of the mushroom ethanolic extracts and of the cosmeceutical formulations (MIC values, mg/mL).

Bacteria Strains	Mushroom Extracts			Cosmeceutical Formulations		
	*A. bisporus*	*P. ostreatus*	*L. edodes*	*A. bisporus* Cream	*P. ostreatus* Cream	*L. edodes* Cream
Gram-positive						
*Enterococcus faecalis*	>20	10	5	>200	200	100
Methicillin sensitive *Staphylococcus aureus*	10	2.5	2.5	200	50	50
Methicillin resistant *Staphylococcus aureus*	10	2.5	2.5	200	50	50
Gram-negative						
*Escherichia coli*	>20	>20	>20	>200	>200	>200
*Pseudomonas aeruginosa*	>20	>20	>20	>200	>200	>200

MIC—minimal inhibitory concentration.

## Supplementary Materials

Supplementary materials can be accessed at: http://www.mdpi.com/1420-3049/21/10/1372/s1.
