# Supplementary material for: Development of Mushroom-Based Cosmeceutical Formulations with Anti-Inflammatory, Anti-Tyrosinase, Antioxidant, and Antibacterial Properties
Source: Molecules. 2016 Oct 14;21(10):1372. doi: 10.3390/molecules21101372 (PMC6274557; doi:10.3390/molecules21101372)
Supplement: Supplementary file 1 [file molecules-21-01372-s001.pdf]

Supplementary Materials: Development of Mushroom-Based Cosmeceutical Formulations with Anti-Inflammatory, Anti-Tyrosinase, Antioxidant and Antibacterial Properties

Oludemi Taofiq, Sandrina A. Heleno, Ricardo C. Calhelha, Maria José Alves, Lillian Barros, Maria Filomena Barreiro, Ana M. González-Paramás and Isabel C. F. R. Ferreira

Table S1. Antibiotic resistance profiles of Gram negative bacteria; MIC values (μg/mL).

Antibiotics	*Escherichia coli*		*Pseudomonas aeruginosa*	
Ampicillin	>8	R		na
Amoxicillin/Clavulanic Acid	≤8/4	S		na
Amikacin		na	≤8	S
Cefuroxim	≤4	S		na
Cefotaxim	≤1	S		na
Ceftazidim	≤1	S	>8	R
Norfloxacin	>8	R		na
Levofloxacin		na	>2	R
Ciprofloxacin	>1	R	>1	R
Nitrofurantoin	≤32	S		na
Fosfomycin	≤16	S		na
Colistin	≤2	S	≤4	S
Gentamicin	>4	R	>4	R
Imipenem		na	>8	R
Meropenem		na	>8	R
Piperacillin/Tazobactam		na	>16	R
Trimethoprim/Sulfamethoxazol	>4/76	R		na
Tobramycin		na	>4	R

S—Susceptible; I—Intermediate; R—Resistant (this classification was made according to the interpretative breakpoints suggested by Clinical and Laboratory Standards Institute—CLSI and European Committee on Antimicrobial Susceptibility Testing (EUCAST); na—not applicable.

Table S2. Antibiotic resistance profiles of Gram positive bacteria; MIC values (μg/mL).

Antibiotics	MRSA		MSSA		*Enterococcus faecalis*	
Penicillin	>8	R	≤0.12	S		na
Ampicillin		na		na	≤4	S
Oxacillin	>0.25	R	≤0.25	S		na
Clindamycin		na	>0.5	R		na
Erythromycin		na	>2	R		na
Ceftarolin	≤1	S		na		na
Gentamicin		na	≤1	S		na
Ciprofloxacin		na	>1	R		na
Levofloxacin		na	>2	R		na
Nitrofurantoin		na		na	≤64	S
Linezolid	≤4	S		na		na
Trimethoprim/Sulfamethoxazol		na	≤2/38	S		na
Vancomycin	≤2	S	≤2	S	≤2	S

MSSA—methicillin-sensitive *Staphylococcus aureus;* MRSA—methicillin-resistant *Staphylococcus aureus;* S—Susceptible; I—Intermediate; R—Resistant (this classification was made according to the interpretative breakpoints suggested by Clinical and Laboratory Standards Institute—CLSI) and European Committee on Antimicrobial Susceptibility Testing (EUCAST).
